# Severe atypical skin disease in two patients with CLL/SLL after BTKi treatment - a case report and literature review

**DOI:** 10.3389/fonc.2024.1467891

**Published:** 2024-12-10

**Authors:** Jingxin Zhou, Wentong Ma, Na Hu, Yuhan Ma, Huayuan Zhu, Ling Gao

**Affiliations:** ^1^ Department of Hematology, The Affiliated Suqian First People’s Hospital of Nanjing Medical University, Suqian, Jiangsu, China; ^2^ Department of Critical Care Medicine, The Affiliated Suqian First People’s Hospital of Nanjing Medical University, Suqian, Jiangsu, China; ^3^ Department of Hematology, The First Affiliated Hospital of Nanjing Medical University, Jiangsu Province Hospital, Nanjing, China

**Keywords:** adverse reaction, CLL/SLL, atypical skin lesion, BTK inhibitor, case report

## Abstract

Dermatological adverse events (AEs) are generally mild during therapy with Bruton’s tyrosine kinase inhibitor (BTKi), and it is often unnecessary to adjust the BTKi dosage or discontinue treatment. However, in this study, we present the cases of two patients diagnosed with chronic lymphocytic leukemia/small lymphocytic lymphoma (CLL/SLL) who experienced severe dermatological AEs during BTKi treatment and subsequently had to discontinue it. The first patient, who previously suffered from rashes, experienced rashes again along with fever when exposed to BTKi. The subsequent dermatological biopsy revealed necrotizing vasculitis. The second patient suffered from skin ulcers concurrently with cough and fever. The BTKi treatment was permanently discontinued when the histopathological biopsy revealed a fungal infection. Therefore, clinicians should pay attention to atypical rashes during BTKi treatment and skin biopsies are necessary for further diagnosis and intervention.

## Introduction

CLL/SLL is the most common type of leukemia in Western countries, with a median diagnosis age of 72 years old (1). The clinical approval of BTKi such as ibrutinib and zanubrutinib has led to a change in CLL/SLL treatment from chemoimmunotherapy to targeted therapy without chemotherapy. The first BTKi long-term follow-up with first-treatment patients had an objective response rate (ORR) of 84%-92%, while the ORR for relapsed and refractory patients was 87%-89%. Moreover, the CLL/SLL patients who used ibrutinib experienced longer progression-free survival (PFS) than those who underwent conventional chemoimmunotherapy. The PFS of the first-treatment patients was eight years, while that for relapsed patients was 52months (2–4). However, research from around the world has revealed that ibrutinib has been partially withdrawn as a clinical treatment due to its potential AEs, which include atrial fibrillation, bleeding, infection, and rash (5, 6). Second-generation zanubrutinib exhibits higher selectivity towards BTK targets, has reduced off-target effects, and leads to fewer AEs such as atrial fibrillation than ibrutinib (7, 8). Regarding skin disorders, most patients experience level 1-2 AEs, while level 3 AEs are occasionally reported (9). For a majority of patients, the condition is self-limiting or can be relieved with local symptomatic treatment. However, approximately 5% of patients require dose reduction or even cessation of treatment (10, 11). Drug-induced rashes can present in a variety of manifestations, often involving complex mechanisms that have not received adequate clinical attention. This article presents two cases of patients with CLL/SLL who developed distinct types of skin lesions while undergoing BTKi treatment, resulting in the eventual discontinuation of the medication.

## Case presentation

Case 1: In 2016, a 54-year-old man with an elevated white blood cell count was admitted to a local hospital to improve his bone marrow morphology and make an immune classification diagnosis for CLL. During the last quarter of 2017, he was given two bottles of chlorambucil.

**Figure 1:**
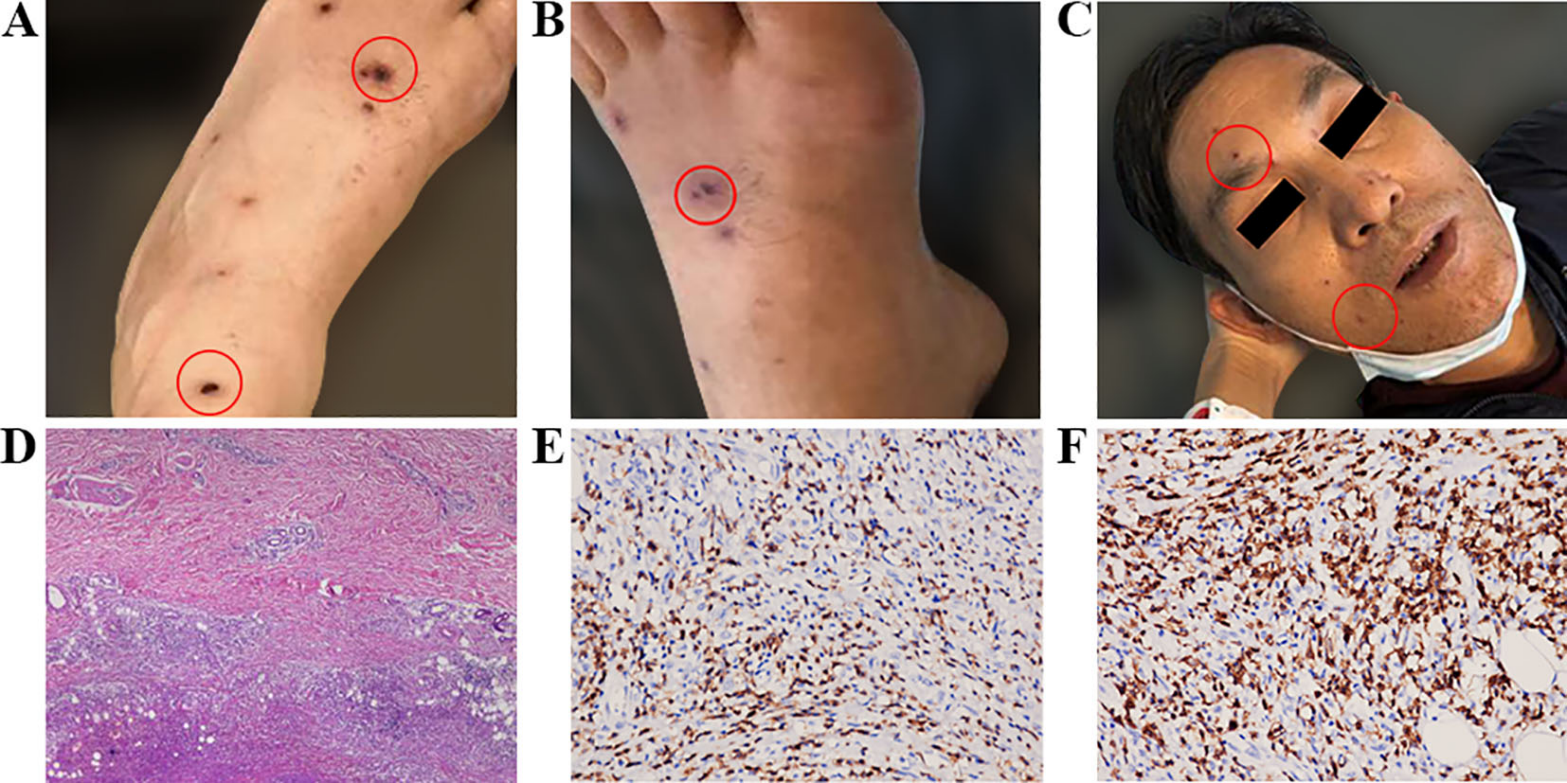
**(A)** Maculopapules appeared soon after the patient first took ibrutinib in 2019; **(B, C)** Maculopapules appeared all over the body when the patient took ibrutinib again in 2020. **(D)** Pathological examination of a skin biopsy on the right calf showed necrotic lesions in the deep dermis and adipose tissue. Lymphocyte infiltration was observed around the necrotic lesions, accompanied by tissue cell reactions. **(E)** CD3 expression detected by immunohistochemical staining on the tissue sections (immunoperoxidase, ×200). **(F)** CD5 expression detected by immunohistochemical staining on the tissue sections (immunoperoxidase, ×400).

By October 2019, the hemoglobin (Hb) and platelets (PLT) levels had decreased considerably. As a result of this treatment indication, he was offered 420 mg ibrutinib once daily. After a month, the patient presented with a maculopapular rash, characterized by polymorphic erythematous papules and macules of varying sizes, predominantly distributed across the trunk and extremities. These lesions are discrete, with well-defined borders, and the patient reported a mild sensation of itching. According to the CTCAE 5.0 (Common Terminology Criteria for Adverse Events) the severity of the rash is graded as Level 1 (**Figure 1A**). Ultimately, the maculopapular rash disappeared after the patient stopped taking ibrutinib. However, the patient did not receive further treatment for his blood disease until May 2020. A blood routine in our hospital in May 2022 revealed an absolute lymphocyte count (ALC) of 166.57×10^9^/L, an Hb count of 76 g/L, and a PLT count of 80×10^9^/L. The peripheral blood immune subtypes were: CD5+, CD10-, CD23-, CD200dim, FMC7dim, CD22dim, and CD79b dim Royal Marsden Hospital (RMH) score 3. Fluorescence *in situ* hybridization (FISH) revealed p53, ATM, 13q14, +12 negative, CCND1/IgH negative, β2-microglobulin 5.8 mg/L (reference value ≤ 2.8 mg/L), and IGHV mutation. Thus, the patient was diagnosed as CLL Rai IV Binet C (CLL-IPI score 3, intermediate). Since the patient had treatment indications due to anemia, thrombocytopenia, and splenomegaly (14 cm below the rib), ibrutinib combined with FCR (fludarabine, cyclophosphamide, and rituximab) was chosen as the first-line treatment.

The patient started taking 420 mg once daily ibrutinib on May 19, 2020. Within the first week, scattered papules and oral blood blisters gradually appeared in the lower limbs, and the PLT count decreased to 28×10^9^/L, signifying a grade 3 AE. Subsequently, the ibrutinib dosage was reduced to 280 mg once daily. The bleeding and rash did not improve, so the dosage was further reduced to 140 mg once daily. The number of skin spots and papules continued to gradually increase until it covered 42% of the entire body (face, lower limbs, and forearms), characterized by polymorphic erythematous papules. These lesions are discrete, with well-defined borders, and the patient reported a mild sensation of itching. According to the CTCAE 5.0, the severity is graded as Level 3 (**Figures 1B, C**). Topical use of calamine lotion was ineffective against the rash. The patient then developed a high fever with a peak temperature of 39°C on July 1. A chest Computerized Tomography (CT) revealed chronic inflammation in both lungs, but pathogen tests for bacteria, fungi, and tuberculosis were negative. After anti-infection treatment, the body temperature did not fall and the rash did not significantly subside. Thus, we believed that there was a correlation between the above conditions and the ibrutinib treatment. A skin biopsy was taken and the pathology presentation revealed necrotic lesions in the deep dermis and adipose tissue of the right calf. Besides, there was lymphocyte infiltration around the necrotic lesions accompanied by tissue cell reactions. The immunohistochemistry results were: CD20 (individual+), CD79a (minority+), PAX5 (individual+), OCT2 (individual+), CD3 (+), CD5 (+), PD1 (-), CD21 (-), CD23 (-), CD10 (-), MUM1 (-), BCL6 (-), BCL2 (minority+), CD30 (-), CD38 (-), CD43 (+), CD68 (tissue cell+), CD163 (tissue cell+), CyclinD (-), Ki67 (approximately 5%+). *In situ* hybridization revealed an Epstein-Barr encoding region (EBER) of (+-). The infiltrating lymphocytes mainly expressed T-lymphocyte markers, suggesting drug-induced vasculitis (DIV) (**Figures 1D–F**). Consequently, we halted ibrutinib therapy on July 1. After seven days of treatment with 3 mg once daily dexamethasone, his temperature returned to normal and his rash disappeared by the end of July. Thereafter, the chemotherapy regimen transitioned to FCR along with rituximab, administered every 28 days. There were no non-hematological adverse events reported during the chemotherapy period. After three rounds of treatment, a midterm evaluation was conducted based on the 2018 International Workshop on Chronic Lymphocytic Leukemia (iwCLL) efficacy assessment criteria, revealing that the patient achieved partial remission (PR), and the readings for minimal residual disease (MRD) in the peripheral blood and bone marrow flow cytometry were negative (<10-4). Subsequently, the patient completed six cycles of FCR chemotherapy, with MRD remaining negative. The current status indicates that the treatment has been discontinued.

Case 2: A 50-year-old male patient was diagnosed with small lymphocytic lymphoma (SLL) by local lymph node biopsy in 2014 due to cervical lymph node enlargement. Subsequently, from January to August 2014, the patient was given one course of COP (cyclophosphamide, vincristine, and prednisone) plus five courses of CHOP (cyclophosphamide, doxorubicin, vincristine, and prednisone). Then, six courses of the FC (fludarabine, cyclophosphamide) regimen were administered between April and September 2015. The patient felt fatigue accompanied by night sweats in July 2016. An enhanced CT scan in our hospital revealed whole body multiple superficial lymph node enlargement, with a maximum lymph node diameter of 2.5 cm. Additionally, IGHV revealed no mutations, while FISH exposed ATM, 13q14 positive. As a result, the patient was diagnosed with SLL stage IV group B, with a physical fitness score of 1 point. According to his treatment indications, he was enrolled in the BGB-3111-205 clinical trial, which was a single-arm, open-label, multicenter, phase II clinical study evaluating the efficacy and safety of BTKi BGB-3111 in the treatment of recurrent or refractory CLL/SLL.

**Figure 2:**
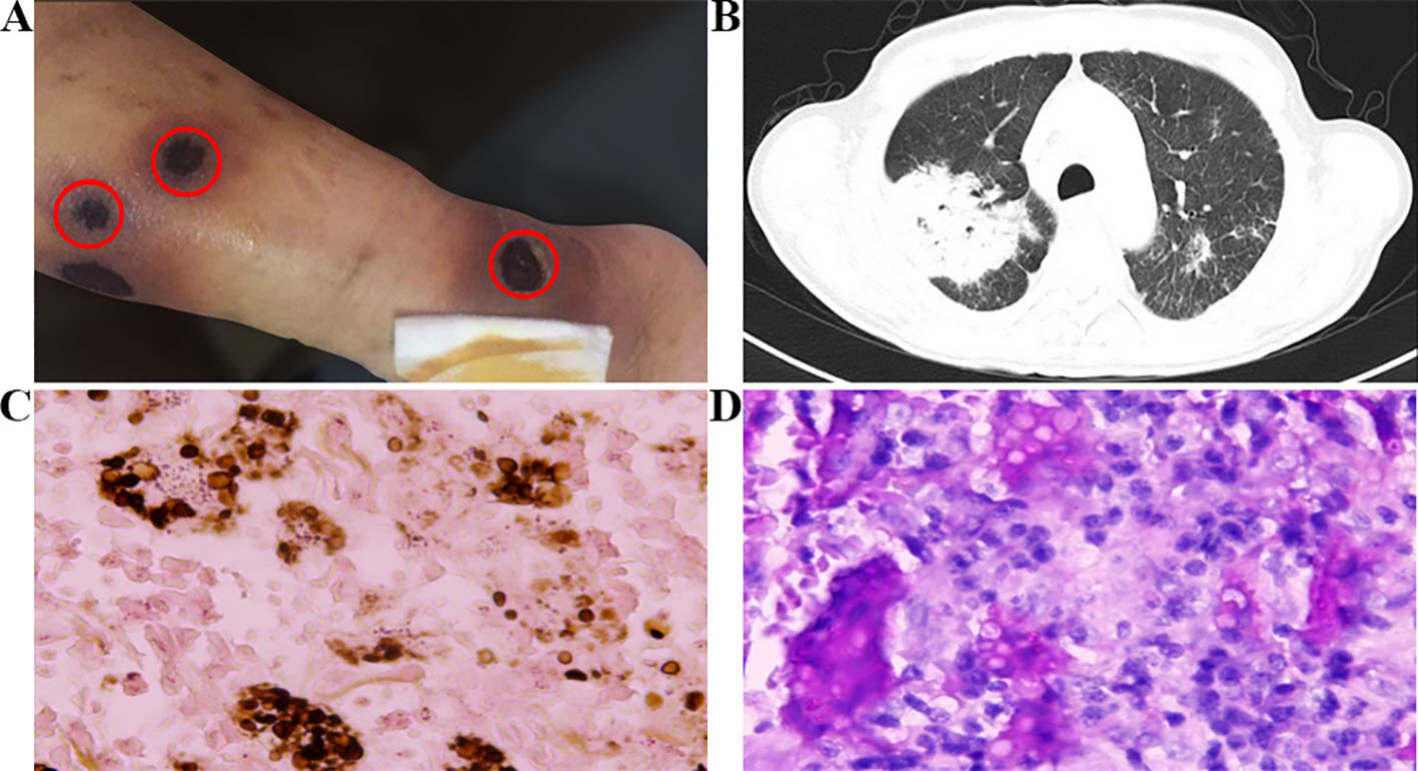
**(A)** Appearance of rash and **(B)** severe pulmonary infection after the use of zanubrutinib. **(C)** Skin biopsy pathology shows positive GMS staining. **(D)** Pathological examination of lung tissue shows positive PAS staining. Original magnification: **(C)** X400; **(D)** X400.

The patient started taking 160 mg twice a day of zanubrutinib (BGB-3111) on July 5, 2017. At the beginning of the treatment, there were no adverse events such as atrial fibrillation, bleeding, or fever. Regular follow-ups were conducted for two months. However, starting from September 3, the patient experienced coughing, white sticky phlegm, and fever, with a peak temperature of 38°C. On September 7, scattered papules with mild itching appeared in both lower limbs. Two days later, the papules on the patient’s skin ruptured in multiple locations, and the ulcers gradually increased in size and depth. Exudation was visible on the surface, and the ulcers reached a maximum diameter of about 2 cm, penetrating deep into the fascia layer. The patient presented with seven skin lesions on the left lower limb, four on the right lower limb, and one on the right upper limb. Comparing this case to the first, the patient initially experienced itching papules which, after two days, progressed to ulceration with the formation of ulcers. These ulcers were surrounded by erythema and exudate, predominantly on the lower limbs, with a scattered distribution that was not continuous, and accompanied by local infection. The CTCAE 5.0 grading is Level 4 (**Figure 2A**). A local hospital chest CT revealed a bilateral lung infection, but there was no significant improvement after local broad-spectrum antibacterial treatment. On September 16, a local blood culture exposed a Candida tropicalis infection. Subsequently, the patient was transferred to our hospital on September 19 for further treatment. Zanubrutinib treatment was stopped and the patient underwent a G-test, which generated a result of 137.1 pg/mL (reference range: 0-100 pg/mL). Besides, a blood culture Galactomannan (GM) test score of 7.178 (reference range: 0-0.5) signified a positive result for Cryptococcus neoformans. A chest CT revealed diffuse multiple consolidation shadows in both lungs, with the right lung being the most prominent, suggesting an infection (**Figure 2B**). Therapeutically, voriconazole was administered intravenously with an initial loading dose of 6mg/kg (300mg twice daily on the first day), followed by a maintenance dose of 4mg/kg (200mg twice daily). On September 21, a skin biopsy was performed, and the pathology revealed diffuse infiltrative and exudative lesions, with the presence of Cryptococcus organisms and capsules, which tested positive for Gomori’s Methenamine Silver Staining (GMS) staining, confirming a diagnosis of Cryptococcal infection (**Figure 2C**). Subsequently, on September 27, a lung biopsy showed chronic inflammation with interstitial fibrosis and some proliferation of alveolar epithelium. A few suspicious Cryptococcus were visible within, alongside a small number of doubly refractive spherical bodies. Special staining with Periodic Acid-Schiff (PAS) was positive, suggesting pulmonary cryptococcosis. Additionally, the capsular polysaccharide antigen of Cryptococcus in the bronchoalveolar lavage fluid also tested positive (**Figure 2D**). It was determined that the patient had Cryptococcus infections in both the skin and lungs and was consequently transferred to the infectious disease department for continued intravenous voriconazole antifungal treatment for a total of 10 days (from September 20th to September 30th), after which their condition improved, and they were discharged. Subsequently, oral fluconazole antifungal (200mg one daily) treatment was initiated upon discharge. However, the patient did not resume oral administration of zanubrutinib and was consequently discharged from the clinical trial group. Moreover, further treatment for SLL was not administered. By February 2018, the skin ulcers on both lower limbs had disappeared, and the patient made the decision to discontinue fluconazole treatment in July 2018, after a total duration of 10 months. Unfortunately, the patient succumbed to a lung infection in June 2019.

## Discussion and literature review

The advent of BTKi has marked a significant advancement in the treatment of CLL and other B cell malignancies. Ibrutinib, the first-generation inhibitor, functions by forming irreversible covalent bonds with BTK, a non-receptor tyrosine kinase belonging to the TEC (transient erythroblastopenia of childhood) family that plays a crucial role in the B-cell receptor signaling pathway. Dermatological toxicities are prominent among the adverse effects of ibrutinib (12). The most common symptom is an increase in nail and hair hardness, with reports of nail cracking and detachment (13). In previous clinical studies, the incidence of rash was between 13-27%, and it could be divided into two types. The first type is asymptomatic petechiae accompanied by thrombocytopenia. Grade 1-2 AEs are more common, while skin and mucosal manifestations are absent or unrelated to allergic reactions. This type of AE is reversible, does not affect BTKi use, and does not require dose adjustment. The other type of rash is purple papules with itching, similar to those that occur in leucocytoclastic vasculitis. These are associated with allergic reactions and appear earlier than the former, with varying degrees of severity. BTK inhibitor-induced drug-related necrotizing vasculitis and leukocytoclastic vasculitis (LCV) exhibit differences in clinical presentation, pathological characteristics, and treatment approaches. Necrotizing vasculitis usually affects small to medium-sized blood vessels, characterized by inflammation and vascular wall necrosis. On the other hand, LCV is an immune complex-mediated small vessel vasculitis, characterized by neutrophil infiltration and nuclear fragmentation. The clinical manifestations of necrotizing vasculitis may include skin damage, joint pain, kidney involvement, and neurological symptoms, while LCV mainly presents with skin symptoms such as purpura and rashes, especially in the lower limbs. The treatment for necrotizing vasculitis may involve immunosuppressants like corticosteroids and cyclophosphamide, along with targeted therapy for the underlying cause. In contrast, treating LCV often requires discontinuing the causative drug, using corticosteroids, and employing other immunosuppressants when needed. The prognosis for LCV is generally favorable, particularly when the causative drug is promptly discontinued and appropriate treatment is administered (14, 15). The toxic side effects of BTKi on the skin include skin infections such as Staphylococcus aureus (9–11), superficial fungi, herpes zoster, reactivation infections of the lip herpes virus, and opportunistic infections caused by invasive fungi.

The first patient in this study developed skin papules after starting a course of ibrutinib for the first time. He stopped taking the medication, but after a 7-month interval, he began taking ibrutinib again. The papules quickly reappeared and there was a grade 3 AE reduction in PLT. After reducing the ibrutinib dose, the PLT level returned to 60×10^9^/L. However, the papules progressively worsened, covering up to 42% of the body surface area at one point. According to CTCAE 5.0, the rash was a grade 3 AE. Furthermore, a non-infectious fever also occurred. After a pathological skin biopsy, disease infiltration and infection were ruled out, and necrotizing vasculitis was confirmed. Subsequent treatment with dexamethasone was extremely effective, and after discontinuing ibrutinib and switching to chemoimmunotherapy, no similar skin lesions were again observed. Thus, we hypothesized that the maculopapules were related to ibrutinib use. Similar cases have been reported in existing literature and pathological biopsies have confirmed that Th1 infiltration predominates in the inflammatory environment (16, 17). This is mainly due to the action of ibrutinib on ITK, which is homologous to BTK in helper T-cells. Subsequently, CD4+T cells polarize toward the Th1 type, thereby inhibiting Th2 immune response and promoting Th1 immune response (18). Other studies have confirmed that the non-ecchymosis-like rash caused by ibrutinib is similar to that caused by epidermal growth factor receptor (EGFR) inhibitors. The EGFR is mainly expressed in the keratinocytes of the epidermis, and EGFR inhibitors promote the release of various cytokines from epidermal cells through the ERK1 and ERK2 pathways. This stimulates the recruitment and activation of neutrophils, monocytes, and lymphocytes, and enhances local inflammatory reactions (19). Ibrutinib inhibits the EGFR due to its off-target effect, which promotes the above-mentioned adverse reactions.

The second patient in this study developed fever, cough, and sputum accompanied by progressive rash after taking zanubrutinib orally for two months. Cryptococcus infection was considered through lung and skin biopsies. Upon discontinuation of zanubrutinib and initiation of antifungal medication, the rash gradually improved. Thus, according to CTCAE 5.0, a grade 4 AE associated with zanubrutinib-related pulmonary and skin infections was suspected. Research has shown that the high-risk factors of BTK inhibitors that cause AEs include the reduction of IgG and IgA levels (20, 21), neutropenia, use of corticosteroids (22). A history of multi-line chemotherapy, and a history of exposure to birds. In July 2017, we determined that the neutropenia level was 1.52×10^9^/L, while the levels of IgG and IgA were significantly reduced, at 4.07 g/L (reference range: 7.5-15.6 g/L) and 0.29 g/L (reference range 0.46-3.04 g/L), respectively. In September 2017, the absolute count and proportion of the CD4+T cells, CD8+T cells, and NK cells were significantly lower, with values of 0.19×10^9^/L (4.8%), 0.21×10^9^/L (5.2%), and 0.08×10^9^/L (2.1%), respectively. This suggests that the patient was in an immunosuppressive state and was more prone to invasive fungal infections. Previous studies have shown that mice with target BTK deficiencies are susceptible to invasive fungal infections (23, 24), mostly due to the off-target inhibitory effect of BTK inhibitors on immune cell function. This interferes with helper T-cell differentiation, inhibits NK cell cytotoxicity, affects neutrophil function and differentiation, acts on macrophages through the TLR9-BTK-NFAT pathway, and reduces macrophage phagocytosis (25). Since neutrophils and macrophages are two important lines of defense in the intrinsic immune response to the aspergillus fungus (26), the off-target effect of BTK inhibitors increases the risk of infection through various mechanisms.

Some existing studies have reported cases where medication was stopped due to rashes caused by Staphylococcus aureus infection (27). In this paper, we described two cases of BTKi treatment that resulted in early atypical skin lesions and ultimately led to permanent discontinuation of the medication. One case was vasculitis characterized by maculopapules that recurred after the use of ibrutinib. The atypical skin lesions subsided one month after the discontinuation of the medication. For grade I-II rashes, topical corticosteroids can still be used. However, rashes of grade III and above require a reduction in dose or temporary discontinuation of both oral antihistamines and systemic corticosteroids (11, 16). The other case we assessed involved invasive fungal skin infections with high-risk factors and the rash resolved four months after discontinuing the BTKi. Previous studies have shown a growing incidence of opportunistic infections caused by invasive fungi. These infections often occur within the first six months of treatment and can present with atypical symptoms. Common fungal infections include Pneumocystis pneumoniae (PJP), Cryptococcus neoformans, and filamentous fungi like aspergillus. Prior to administering BTKi small molecule targeted drugs, clinicians must thoroughly assess patients for potential infection risk. This evaluation should encompass disease status, overall physical health, underlying medical conditions, potential drug interactions, and any relevant contact history (25). Pathological confirmation has linked both cases to skin lesions and side effects of BKTi. These cases provide a crucial learning opportunity for clinicians to comprehensively grasp the complexities of BTKi treatment and enhance patient care by promptly identifying and addressing skin reactions, which have a significant impact on patient quality of life and treatment adherence. However, it is important to acknowledge the limitations of our report. The small number of cases and inherent bias and data incompleteness affect the generalizability of the findings. And the relatively short follow-up period constrains the understanding of long-term outcomes and potential late complications. Future studies with larger sample sizes and longer follow-up periods are needed to better comprehend similar cases and their implications.

During BTKi treatment, it is essential to carefully monitor adverse events. Fortunately, early onset atypical rash can be easily diagnosed through skin pathological biopsy. Patients who stop taking BTKi can either choose highly selective second-generation BTK inhibitors or other targeted drugs.

## Data Availability

The original contributions presented in the study are included in the article/supplementary material. Further inquiries can be directed to the corresponding authors.
